# Association of Hyperlipidemia and Cardiovascular-Kidney-Metabolic Risk Factor Severity With Incidental Coronary Artery Calcium

**DOI:** 10.1016/j.jacadv.2026.103157

**Published:** 2026-08-18

**Authors:** Abigail S. Pan, Sui Zhang, Seth S. Martin, Neha J. Pagidipati, Michael Shapiro, Roger S. Blumenthal, Janani Rangaswami, Carl E. Orringer, Chiadi E. Ndumele, Jelani K. Grant

**Affiliations:** a Johns Hopkins School of Medicine, Baltimore, Maryland, USA; b Welch Center for Prevention, Epidemiology and Clinical Research, Johns Hopkins University, Baltimore, Maryland, USA; c Johns Hopkins Ciccarone Center for the Prevention of Cardiovascular Disease, Baltimore, Maryland, USA; d Duke Clinical Research Institute, Durham, North Carolina, USA; e Department of Cardiovascular Medicine, Wake Forest University Health Sciences, Winston-Salem, North Carolina, USA; f Division of Nephrology, Department of Medicine, Washington DC VA Medical Center, Washington, District of Columbia, USA; g The George Washington University School of Medicine and Health Sciences, Washington District of Columbia, USA; h Naples Comprehensive Health System, Naples, Florida, USA; i Division of Cardiovascular Medicine, Department of Medicine, Perelman School of Medicine at the University of Pennsylvania, Philadelphia, Pennsylvania, USA

**Keywords:** cardio-kidney, cardiometabolic health, coronary artery calcification (CAC), prevention, risk factors

**What** **is the clinical question being addressed?**

What is the burden and degree of severity of hyperlipidemia and CKM risk factors among adults with incidentally identified CAC?

**What is the main finding?**

Although the prevalence of severe hyperlipidemia was less with greater incidental CAC, prevalences and severity of diabetes, hypertension, and chronic kidney disease were worse. This suggests that although hyperlipidemia may be more aggressively controlled in those with incidental CAC, broader CKM risk is likely underaddressed, representing unmet potential for enhancing cardiovascular prevention.

Coronary artery calcium (CAC) on electrocardiography (ECG)-gated cardiac computed tomography (CT) strongly predicts atherosclerotic cardiovascular disease.^1^ CAC incidentally identified on non–ECG-gated chest CT (“incidental CAC”) carries similar prognostic significance to ECG-gated scans, with moderate-to-severe incidental CAC typically corresponding to a CAC score ≥100 Agatston units, a guideline-based threshold to consider statin initiation.^1^ Although lipid-lowering therapy is emphasized for individuals with elevated CAC,^1^ CAC reflects cumulative exposure to multiple cardiovascular-kidney-metabolic (CKM) risk factors.^2^ The extent to which incidental CAC indicates an imperative to address CKM risk factors beyond hyperlipidemia remains unclear.

## Methods

**Figure 1:**
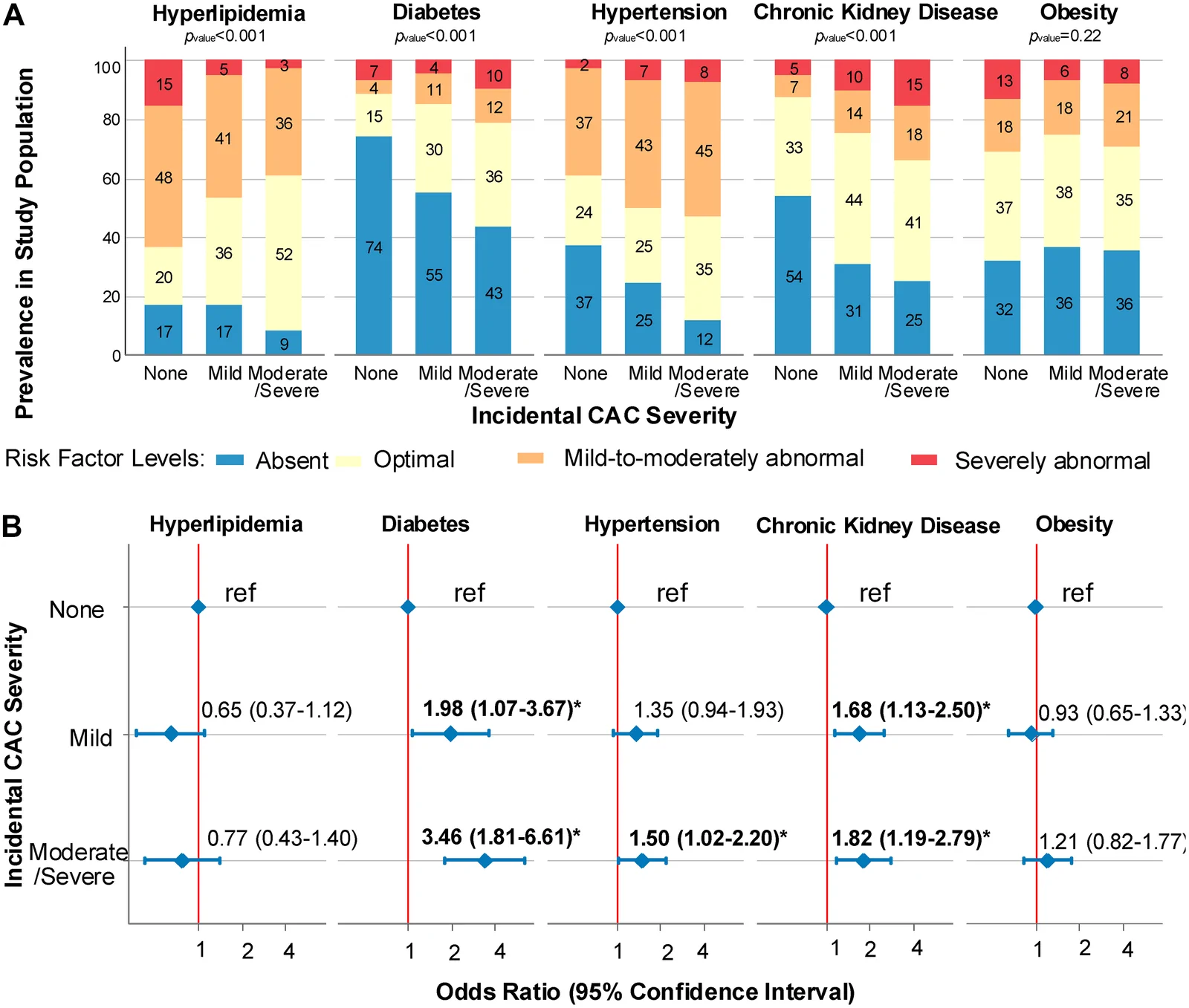
Hyperlipidemia and CKM Risk Factor Severity by Incidental CAC (A) Stacked bar graphs demonstrating distribution of risk factor severity categories by incidental CAC severity. Prevalent hyperlipidemia was defined by documented diagnosis during chart review, use of any lipid-lowering therapy, low-density lipoprotein cholesterol (LDL-C) ≥100 mg/dL, or non–high-density lipoprotein cholesterol (non-HDL-C) ≥130 mg/dL. Prevalent CKM risk factors were defined using standard clinical criteria: obesity (body mass index ≥30 kg/m^2^), diabetes (prior clinical diagnosis or HbA1c ≥6.5%), hypertension (prior clinical diagnosis or BP ≥130/80 mm Hg), and chronic kidney disease (eGFR <60 mL/min/1.73 m^2^). (B) Adjusted ordinal regression analyses evaluating the association between CAC severity and the odds of worse risk factor severity (ORs >1 reflect higher odds of being in a more severe CKM risk category relative to no incidental CAC). ∗*P* values calculated using the chi-square test, assessing for differences in categories of risk factor severity across incidental CAC levels. BP = blood pressure; CAC = coronary artery calcium; CKM = cardiovascular-kidney-metabolic; eGFR = estimated glomerular filtration rate; HbA1c = glycosylated hemoglobin; OR = odds ratio; ref = Reference.

We conducted a cross-sectional analysis of 745 adults drawn from 2 1-month consecutive convenience samples collected in 2019 and 2021 as part of a quality improvement initiative at an academic medical center in Florida (previously described),^3^ all of whom underwent non–ECG-gated, noncardiac chest CTs. Incidental CAC was visually categorized as none, mild, or moderate-to-severe by 2 board-certified chest radiologists. Across incidental CAC categories, we used logistic regression (ie, adjusting for age, sex, race, and smoking) to estimate odds of hyperlipidemia and the CKM risk factors of obesity, type 2 diabetes mellitus (T2D), hypertension, and chronic kidney disease (CKD), defined using the standard criteria (Figure 1). We used established thresholds to categorize risk factor severity as absent, optimal, mild-to-moderately abnormal, and severely abnormal across incidental CAC categories: hyperlipidemia severity (ie, absent, optimal, mild-to-moderately abnormal and severely abnormal) was defined by low-density lipoprotein cholesterol/non–high-density lipoprotein cholesterol thresholds of <100/<130 mg/dL, <100/<130 mg/dL with prior hyperlipidemia or lipid-lowering therapy, 100–<160/130–<190 mg/dL, and ≥160/≥190 mg/dL, respectively. Hypertension severity was defined by blood pressure <130/80 mm Hg without hypertension, systolic blood pressure (SBP) <130 mm Hg with hypertension, SBP 130–<160 mm Hg, and SBP ≥160 mm Hg. Diabetes severity was defined by glycosylated hemoglobin (HbA1c) <6.5%, diabetes with HbA1c <7% or no diabetes with HbA1c ≥6.5%, HbA1c 7%–<8%, and HbA1c ≥8%, respectively. Obesity severity was defined by body mass index 18.5–<25 kg/m^2^, 25–<30 kg/m^2^, 30–<35 kg/m^2^, and ≥35 kg/m^2^, respectively. Because measurements of urinary albumin were unavailable, CKD severity was defined using estimated glomerular filtration rate alone as ≥90 mL/min/1.73 m^2^, 60–<90 mL/min/1.73 m^2^, 45–<60 mL/min/1.73 m^2^, and <45 mL/min/1.73 m^2^, respectively. After confirming the proportional odds assumption was met, we used ordinal logistic regression (adjusted for age, sex, race, and smoking) to estimate the odds of being in a worse risk factor severity category in association with mild and moderate-to-severe incidental CAC vs no incidental CAC.

This study was approved by the Institutional Review Board. Informed consent was waived for retrospective data analyses. Analyses were performed using Stata, version 18.0 (StataCorp LLC). Two-sided values of *P* < 0.05 were considered statistically significant.

## Results

Among 745 adults (mean age: 64 years, 52% women, 54% Hispanic), incidental CAC was classified as none in 39%, mild in 28%, and moderate-to-severe in 33% of individuals. Comparing those with none, mild, and moderate-severe incidental CAC, there was a similar hyperlipidemia prevalence (83%, 83%, and 91%; *P* = 0.13), but a progressive increase in the prevalence of CKM risk factors including T2D (16%, 29%, and 35%; *P* < 0.001), hypertension (62%, 75%, and 88%; *P* < 0.001), and CKD (38%, 58%, and 68%; *P* < 0.001). Obesity prevalence did not differ across incidental CAC categories (*P* = 0.33).

In multivariable-adjusted analyses, compared with no CAC, moderate-to-severe incidental CAC was associated with a nonsignificant increase in the odds of hyperlipidemia (OR: 2.08; 95% CI: 0.78-5.58), but higher odds of T2D (OR: 2.40; 95% CI: 1.46-3.95), hypertension (OR: 2.95; 95% CI: 1.75-5.00), and CKD (OR: 1.76; 95% CI: 1.15-2.71). There was no significant association between CAC category and the odds of obesity.

In terms of risk factor severity (Figure 1A), hyperlipidemia severity improved with increasing CAC severity, with worse than optimal hyperlipidemia decreasing from 63% among those with no CAC to 39% among those with moderate-to-severe CAC (*P* < 0.001). This corresponded with higher statin use in those with no CAC vs moderate-to-severe CAC (23% vs 65%). In contrast, from no CAC to moderate-to-severe CAC, the prevalence of worse than optimal diabetes, hypertension, and CKD increased from 11% to 22%, 39% to 53%, and 12% to 33%, respectively (all *P* < 0.001). No significant differences in obesity severity were seen across CAC levels (*P* = 0.22).

In ordinal regression (Figure 1B), compared to no CAC, higher CAC was not significantly associated with hyperlipidemia severity category. In contrast, moderate-severe CAC was associated with a greater likelihood of being in a worse severity category for T2D (OR: 3.46; 95% CI: 1.81-6.61), hypertension (OR: 1.50; 95% CI: 1.02-2.20), and CKD (OR: 1.82; 95% CI: 1.19-2.79), vs no CAC. Findings were similar when individuals with prevalent atherosclerotic cardiovascular disease (11%) or no prior T2D diagnosis were excluded.

In this cross-sectional analysis, the prevalence and odds of most CKM risk factors were higher with greater incidental CAC severity. Although the prevalence of more severe hyperlipidemia was less with increasing CAC severity, the prevalence of worse than optimal CKM risk factors—particularly T2D, hypertension, and CKD—was higher. The odds of worse CKM risk factor severity were higher among individuals with more severe incidental CAC.

## Discussion

These findings align with current guideline-based management, in which elevated CAC is an indication for intensified lipid-lowering therapy due to higher absolute cardiovascular disease risk.^1^ Our results suggest that hyperlipidemia is possibly being more aggressively addressed in individuals with greater incidental CAC, as reflected by higher statin use. However, our findings demonstrate the opposite pattern for CKM risk factors, with greater severity of T2D, hypertension, and CKD found among those with more incidental CAC. Shared pathophysiologic pathways across CKM conditions contribute to accelerated atherosclerosis, including inflammation, endothelial dysfunction, and oxidative stress, with CAC representing cumulative exposure to these processes across multiple risk domains.^2^ Relatedly, modeling studies suggest the potential of CAC for informing preventive strategies beyond lipid lowering, including aspirin use, hypertension management, and glucagon-like peptide-1 receptor agonist use.^4^ Although obesity was not associated with greater CAC severity in our analysis, prior studies have shown conflicting findings regarding whether the association of obesity with coronary artery disease is independent of metabolic risk factors.^5^ Furthermore, our use of body mass index may not adequately capture central adiposity and its cardiometabolic consequences.

A key implication of this work is that suboptimal CKM risk factors represent a gap in comprehensive preventive care among those with coronary atherosclerosis by imaging. Individuals with moderate or greater incidental CAC may benefit from systematic evaluation for CKM risk factors and comprehensive risk factor optimization. Further cardiovascular risk reduction can be achieved by utilizing CKM therapies with multisystem benefits including glucagon-like peptide-1 receptor agonists, sodium-glucose cotransporter 2 inhibitors, renin-angiotensin system inhibitors, and mineralocorticoid receptor antagonists, when indicated. With over 10 million non–ECG-gated chest CT scans performed annually in the United States,^1^ incidental CAC represents a scalable opportunity to enhance cardiovascular risk stratification and prevention for high-risk patients.

### Study limitations

The single-center, cross-sectional design may limit generalizability and precludes temporality assessments. Risk factor abnormalities reflect both pathologic severity and clinical treatment. Ascertainment and confounding bias may have impacted results. Nevertheless, this study leverages incidental CAC assessment and detailed characterization of CKM risk factor prevalence and severity.

## Conclusions

Greater incidental CAC severity is associated with a lesser prevalence of worse hyperlipidemia severity, but a greater prevalence and severity of CKM risk factors, particularly T2D, hypertension, and CKD. This highlights incidental CAC as an opportunity to identify high-risk individuals who may benefit from comprehensive CKM-focused prevention beyond lipid lowering alone.

## Funding support and author disclosures

Ms Zhang and Dr Ndumele were supported by grant AHA20SFRN35120152. All other authors have reported that they have no relationships relevant to the contents of this paper to disclose.
